# From Regression to Vision Transformers: A Narrative Review of Predictive Modelling in Dental Implantology and the Gap Between Algorithmic Performance and Clinical Adoption

**DOI:** 10.7759/cureus.114906

**Published:** 2026-08-21

**Authors:** Akash Gopi, Vishwa Deepak Singh

**Affiliations:** 1 Prosthodontics and Crown and Bridge, Teerthanker Mahaveer Dental College and Research Centre, Moradabad, IND; 2 Prosthodontics, Teerthanker Mahaveer Dental College and Research Centre, Moradabad, IND

**Keywords:** clinical adoption, deep learning, dental implantology, dental implants, implant stability quotient, machine learning, prediction models, tripod-ai, vision transformer

## Abstract

Predictive modelling has moved from simple regression equations to deep, image-native architectures capable of localising an implant position on a cone-beam computed tomography (CBCT) scan with sub-millimetre precision. Yet the discipline that builds these models and the clinicians who would use them appear, on current evidence, to occupy different timelines. This narrative review synthesises comparative performance data across traditional statistical, machine learning, and deep learning approaches to dental implant prognostication; situates these methods within the broader literature on prediction-model validation and reporting; and juxtaposes this technical trajectory against field survey data describing how practising dentists actually perceive and use predictive and digital tools. The synthesis suggests that while deep learning architectures now substantially outperform logistic regression and Cox models on discrimination metrics, routine clinical uptake remains constrained less by algorithmic ceiling and more by validation gaps, interpretability concerns, and infrastructural readiness at the chairside.

## Introduction and background

Implant therapy has become a default option for replacing missing teeth, but its long-term success is not uniform: it is shaped by bone quality and quantity, surgical technique, prosthetic loading protocol, and patient-level factors such as smoking, parafunction, and glycaemic control [1,2]. A clinician assessing a candidate for implant placement is, in effect, performing an informal multivariable risk calculation every time they weigh radiographic bone density against a patient's HbA1c reading, their loading plan against the span being restored, and their surgical margin against the patient's compliance history. As these determinants interact in ways that are difficult to weigh consistently from experience alone, and because the consequences of misjudging them range from early failure to costly prosthetic remakes, prediction models have been proposed as a way of formalising a risk assessment that clinicians already perform, however informally, at the chairside.

Over roughly two decades, the methodological toolkit available for this task has expanded enormously. Early efforts relied on odds ratios derived from logistic regression fitted to modest retrospective cohorts; the current generation of tools includes vision transformer networks that read an entire cone-beam computed tomography (CBCT) volume directly and output a recommended three-dimensional implant position without any human-engineered features at all. This trajectory mirrors a broader pattern across medical imaging and clinical prognostication, in which each new architecture generation has, on benchmark datasets, outperformed the last on measures of discrimination. Dental implantology is a particularly instructive case study within this broader movement because it combines a well-defined, binary-adjacent outcome (osseointegration success versus failure, or acceptable versus unacceptable implant position), a rich and increasingly standard three-dimensional imaging modality in CBCT, and a practitioner base that, unlike some medical specialties, spans a very wide range of training backgrounds, from oral and maxillofacial surgeons and prosthodontists in academic centres to general dental practitioners in solo community practice.

This review draws together three threads of evidence that are rarely considered together. The first is a comparative technical appraisal of the prediction model families that have been used for implant prognosis and implant position planning, spanning traditional statistical methods, classical machine learning, and deep learning [3]. The second is a broader methodological account of how such models are developed, validated, and reported across dentistry, drawing on guidance that is increasingly being adopted by indexed prosthodontic and implantology journals [4,5]. The third is empirical survey data describing how dental practitioners in India currently perceive implant longevity, predictive tools, and digital technology adoption in their own practices [6]. Read in isolation, each thread tells a coherent but partial story: the technical literature describes a field of rapidly improving algorithms, the methodological literature describes a discipline still working out how to validate and report those algorithms responsibly, and the survey literature describes a workforce that has, for the most part, not yet encountered any of this in daily practice. The intent of bringing these threads together is not to catalogue every published algorithm in the field, which would be neither feasible nor useful, but to ask a more practical and more uncomfortable question: Does the accuracy gained by moving toward increasingly complex architectures translate into anything a treating clinician, working with the equipment and training they actually have, can use?

## Review

Literature for this narrative review was identified through searches of PubMed/MEDLINE and Scopus, covering publications from January 2020 to July 2026, using combinations of terms including "dental implant", "predictive model", "machine learning", "deep learning", "vision transformer", "CBCT", and "clinical adoption". Reference lists of retrieved articles were also hand-searched for additional relevant sources. Sources were selected for relevance to implant outcome prediction, model validation methodology, or clinician-reported technology adoption, rather than through a systematic or exhaustive screening process, consistent with the narrative (rather than systematic) design of this review.

Statistical foundations of implant outcome prediction

Logistic regression remains the most widely reported approach for binary implant outcomes such as osseointegration success or failure, and for good reason. Its appeal lies in interpretability: each predictor's contribution is expressed as an odds ratio with a confidence interval that a clinician can relate directly to a patient sitting in front of them, in a form that maps naturally onto the kind of counselling conversation a consent discussion requires. A clinician can tell a patient, in plain terms, that a given risk factor roughly doubles or halves their odds of a favourable outcome, and can show the arithmetic behind that statement if asked. This transparency is not a minor convenience; in a field where treatment decisions carry direct medico-legal consequences, the ability to trace a recommendation back to an auditable coefficient is often valued as highly as raw predictive accuracy. The principal weakness of logistic regression is its assumption of a linear, additive relationship between predictors and outcome on the logit scale, an assumption that breaks down when biomechanical and biological variables interact non-linearly, as they frequently do when, for example, the effect of poor bone density is compounded rather than merely added to by smoking status or by an unfavourable crown-to-implant ratio [3,7].

Where the outcome of interest is time-to-event rather than binary, for example, time to implant failure rather than failure or success alone, Cox proportional hazards modelling has been the conventional choice, valued for its ability to handle censored follow-up data from patients who are lost to review or who have not yet reached the study's endpoint at the time of analysis. Its central assumption, that the hazard ratio between comparison groups remains constant over time, is frequently violated in practice because surgical technique, loading protocol, and patient compliance rarely stay fixed across a multi-year follow-up period; a risk factor that dominates in the first year of loading, such as primary stability at placement, typically matters far less by the fifth year, by which point peri-implant maintenance and occlusal loading history have become the more influential determinants. This time-varying hazard structure limits the robustness of long-term survival estimates derived from a model that, by design, treats the hazard ratio as fixed [3].

Beyond the choice of model family, the statistical foundations of implant prediction research also depend on how carefully predictors are selected and how the resulting model is calibrated, and these two considerations are frequently under-discussed relative to the headline discrimination statistics a paper reports. Discrimination, typically summarised by the area under the receiver operating characteristic curve, describes how well a model ranks patients relative to one another; it says nothing about whether the absolute probability a model outputs, for example a stated 70% chance of five-year survival, actually corresponds to the observed frequency of survival among patients given that prediction. A model can discriminate well while being poorly calibrated, systematically over- or under-stating absolute risk in a way that would mislead a patient-facing consent conversation even though the model correctly ranks higher-risk patients above lower-risk ones. Calibration plots and calibration slopes are reported far less consistently in the implant prediction literature than discrimination statistics, which is a gap this review returns to in the discussion of methodological considerations below.

Machine learning and deep learning approaches

Ensemble and Kernel-Based Methods

Support vector machines and ensemble tree methods, particularly random forests and, increasingly, gradient-boosted tree variants such as XGBoost, relax the linearity assumption of classical regression and have reported classification accuracies in the region of 85-90% for implant success or failure discrimination on tabular clinical and radiographic feature sets [1,3]. These methods work by partitioning the predictor space into progressively finer regions, or by finding a maximally separating boundary in a transformed feature space, rather than by fitting a single global linear equation, which allows them to capture threshold effects and interactions that logistic regression would otherwise miss unless those interaction terms were specified in advance by the modeller.

Random forests carry a particular advantage for thesis-level and retrospective clinical research, which constitutes a large share of the dental implant prediction literature: they tolerate missing data reasonably well, resist overfitting through bootstrap aggregation across many decision trees, and yield a variable-importance ranking that offers a partial window into which predictors are driving the model, a middle ground between the full transparency of a regression coefficient and the opacity of a deep network's internal representations [3]. This variable-importance output is one reason random forests have become a common default choice for retrospective single-centre implant cohorts, where sample sizes are often too small to support a deep network but where the investigator still wants some indication of which clinical or radiographic factors the model is weighting most heavily. The trade-off is that these ensemble methods generally still require the modeller to hand-engineer the predictor set in advance, whether from chart review, radiographic measurement, or laboratory values, and their performance is therefore bounded by the quality and completeness of that manually curated feature set in a way that image-native deep learning approaches are not.

Radiographic Deep Learning

The most significant accuracy gains in the recent literature come from architectures built specifically to read CBCT and radiographic data directly, without first requiring a human to reduce the image to a handful of measured features. Convolutional neural networks (CNNs) established the baseline for automated detection of bone loss and periapical or peri-implant lesion identification, learning hierarchical spatial filters directly from labelled image data [7,8]. Convolutional architectures, however, are constrained by the local receptive field of their convolutional kernels, which means that early layers of the network only “see” a small neighbourhood of voxels at a time and must build up an understanding of larger structures through depth; this can make it harder for a purely convolutional network to relate a subtle finding in one region of a CBCT volume, such as a thin buccal plate, to contextual information located some distance away, such as the position of the inferior alveolar canal on the opposite side of the arch.

The more recent implant-positioning literature has moved towards vision-transformer and multi-stream architectures that address this limitation directly. Vision transformers replace convolutional filters with a self-attention mechanism that, in principle, allows every region of an image to attend to every other region from the earliest layers of the network, which is particularly well suited to a task like implant position planning where the correct answer in one part of the arch genuinely depends on structures elsewhere in the same volume. ImplantFormer applies a vision-transformer backbone to CBCT volumes to localise optimal implant position, outperforming conventional CNN baselines on positional accuracy by making use of this global attention property [9]. The Two-Stream Implant Position Regression (TSIPR) network pairs a dedicated implant-region detector with multi-scale patch embedding in a second processing stream, an architectural choice intended to manage variability in tooth spacing and bone texture across different patients and different regions of the same arch [10]. The Text-Condition-Embedded Implant Position (TCEIP) regression network goes a step further by fusing textual descriptors of the intended implant site, such as the tooth position being restored, with image features extracted from the CBCT volume, which is particularly useful in multi-tooth edentulous spans where the image information alone is ambiguous about which specific site within the span is being planned [11]. The most recent architecture considered here, TCSloT, extends this line of work further still by incorporating three-dimensional contextual slices around the planned site together with explicit implant angulation awareness, allowing the network to reason jointly about position and trajectory rather than treating them as separate outputs [11].

These successive architectural refinements share a common thread: each generation adds a mechanism for incorporating more contextual information, whether spatial, textual, or angular, into the prediction, and each generation has, on the benchmark datasets reported in its originating paper, outperformed its predecessor. It is worth noting, however, that this steady architectural progression has so far been demonstrated almost entirely on curated research datasets assembled by the groups proposing each new method, a point this review returns to when discussing external validation. The computational requirements of these networks are also non-trivial; training a vision-transformer model on volumetric CBCT data typically requires graphics processing unit hardware and a training dataset of a scale that is realistic for a specialised imaging research group but is well beyond what any individual dental practice or even most dental colleges could assemble independently, which has implications for how such models could realistically be updated, retrained, or locally validated once deployed.

Comparative Performance Across Model Families

Table 1 summarises illustrative accuracy and area under the receiver operating characteristic curve figures reported across the traditional statistical, machine learning, and deep learning families discussed above [3,9-11]. Read as a group, the pattern is a fairly steady stepwise gain moving from regression through ensemble learning to transformer-based radiographic models, with the newest three-dimensional context-aware networks reporting accuracy above 97% and an area under the curve approaching 0.99.

**Table 1:** Illustrative comparative performance of prediction model families applied to dental implant outcomes and cone-beam computed tomography-based implant positioning, compiled from published methodological descriptions. Figures are representative of reported ranges rather than a single pooled meta-analytic estimate. AUROC: area under the receiver operating characteristic curve. Source: [3,9-11].

Model	Accuracy (%)	AUROC	Notable characteristics
Logistic Regression	78.5	0.79	Traditional statistical model; high interpretability, limited with non-linear interactions
Cox Proportional Hazards	76.0	0.77	Time-to-event survival modelling; assumes proportional hazards
Support Vector Machine	85.2	0.88	Effective in high-dimensional feature spaces
Random Forest	88.3	0.91	Ensemble learning; robust to missing data, good variable-importance mapping
ImplantFormer (Vision Transformer-based)	95.2	0.96	Vision-transformer localisation from cone-beam computed tomography
TSIPR (Two-Stream Implant Position Regression network)	96.5	0.97	Dual-stream regression network for implant positioning
TCEIP (Text-Condition-Embedded Implant Position regression network)	97.1	0.98	Text-conditioned cross-modal regression network
TCSloT (Three-dimensional Contextual Slope-aware Implant Positioning Network)	97.8	0.99	Three-dimensional contextual, slope-aware positioning network

Group-level statistical comparison across these three families, traditional statistical models, classical machine learning, and deep learning, shows the difference in mean accuracy to be large and consistent: deep learning architectures separate clearly from both machine learning and traditional statistical approaches, and machine learning in turn separates from traditional regression and Cox modelling, though the margin between machine learning and traditional methods is narrower than the margin between deep learning and either of the other two groups. Pooled area under the curve likewise rises from roughly 0.78 for traditional models to roughly 0.90 for machine learning and roughly 0.98 for deep learning approaches, reinforcing that the largest single gain in discriminative capacity comes from moving to image-native deep architectures rather than from moving between statistical and shallow machine-learning methods that still rely on hand-engineered tabular features [3]. It bears emphasising, however, that these three families are not being compared on identical tasks or identical datasets in most of the underlying literature: the traditional and machine learning figures largely describe binary success or failure classification from tabular clinical predictors, while the deep learning figures largely describe a geometrically different task, namely, continuous implant position regression from volumetric imaging. The apparent stepwise improvement should therefore be read as indicative of a broad trajectory in the field rather than as a like-for-like head-to-head benchmark, a caveat this review develops further in the limitations section.

Methodological considerations: Validation, sample size, and reporting

Accuracy figures of this kind are only as trustworthy as the validation strategy behind them. Internal validation, whether k-fold cross-validation or bootstrap resampling performed on the same population used for training, estimates how a model behaves on data drawn from a similar distribution to its training set, but it is external validation, performed on an independent cohort ideally drawn from a different centre, a different scanner, or a different time period, that is considered the appropriate standard before any claim of generalisability is made. Imaging-based models are especially vulnerable to this distinction because CBCT acquisition parameters, voxel size, field of view, and reconstruction algorithms differ meaningfully between manufacturers, and a model trained on volumes from one CBCT unit and acquisition protocol can lose a substantial share of its reported accuracy when applied to images from a different machine, even when the underlying anatomy and clinical question are identical [4,7].

Sample size planning differs meaningfully by model family and deserves more attention in this literature than it typically receives. Regression-based models are conventionally justified using an events-per-variable heuristic, commonly around 10 outcome events per candidate predictor, to avoid unstable coefficient estimates and overfit odds ratios that would not replicate in an independent cohort [5]. Deep learning models operate under a different, less standardised logic, where sample size requirements scale with model capacity and are usually addressed empirically, through learning curves and data augmentation, rather than through a pre-specified formula; this makes it correspondingly harder for a reader to judge, from a methods section alone, whether a reported deep learning result was obtained from a training set of adequate size or whether it reflects an artefact of a small, homogeneous dataset. For an in vivo comparative design, for instance a trial contrasting conventional implant planning against artificial intelligence-assisted planning using implant stability quotient trajectories and one-year survival as outcomes, sample size estimation should be built around the expected between-arm effect size in implant stability quotient, the anticipated variability of that outcome, and an explicit allowance for dropout, with implant survival treated as an event-based secondary outcome requiring its own, typically larger, sample size check given the low baseline failure rate of modern implant systems [5].

A further methodological issue that is frequently glossed over in this literature is class imbalance. Implant failure, mercifully, is a comparatively rare event in modern practice, which means that a model trained on a typical retrospective cohort will see many more successful outcomes than failed ones. A model can achieve a superficially impressive raw accuracy figure simply by predicting success for almost every case, while performing very poorly on the clinically important minority class of failures; this is precisely why accuracy alone, the metric most consistently reported across the studies summarised in Table 1, is an incomplete measure of model quality, and why sensitivity, specificity, positive and negative predictive values, and threshold-independent measures such as the area under the precision-recall curve deserve to be reported alongside it, particularly for outcomes as imbalanced as implant failure.

Reporting quality is a further determinant of whether a published model can be appraised or reproduced at all. The Transparent Reporting of a multivariable prediction model for Individual Prognosis Or Diagnosis statement (TRIPOD), and its machine learning extension, TRIPOD-AI, provide structured checklists covering predictor selection, model development, and validation methodology, and indexed prosthodontic and implantology journals increasingly expect TRIPOD-AI-consistent reporting as a condition of publication [4]. Missing data handling is a related and frequently under-reported choice within this framework: complete case analysis, in which any patient record with a missing value is simply dropped, is simple to implement but can bias results and needlessly shrink an already modest sample size, whereas multiple imputation is generally preferred when missingness is unlikely to be completely random, as is typically the case when, for example, smoking status or glycaemic control values are missing preferentially from records of patients who attended fewer follow-up visits [5]. Alongside reporting standards, code and data availability remain inconsistent across this literature; several of the deep learning architectures discussed above are described in sufficient methodological detail to be conceptually understood but not necessarily in sufficient detail, absent released code or model weights, for an independent group to reproduce the reported figures exactly, which limits how confidently this review, or any reader, can treat the headline numbers in Table 1 as fixed rather than as best-case figures from the reporting group.

The clinical adoption gap: Evidence from practising dentists

The technical trajectory described above can be set against field survey data describing how dentists in India currently engage with implant-longevity assessment and digital technology. In a cross-sectional questionnaire study of dental professionals conducted at a single institution, the majority of respondents were relatively early in their careers, with the large majority in practice for under five years, and a majority practising in urban settings. Just over half had received no specific implant-related education, and self-rated knowledge of implantology skewed towards the moderate-to-limited range for a substantial share of respondents [6].

Two findings from this survey are particularly relevant to the modelling literature reviewed above. First, despite widespread agreement among respondents that implants are a durable long-term solution, most had not personally placed an implant in the preceding year, and the majority had received no implantology-specific training; the population most likely to benefit from a decision support tool is, on current evidence, least equipped to interpret one critically, and a tool designed and validated by specialists may not transfer well to the case mix and confidence level of this broader, less specialised workforce. Second, uptake of the digital infrastructure that artificial intelligence-assisted planning presupposes was uneven across the surveyed population: digital radiography was common, but intraoral scanners and computer-aided design and computer-aided manufacturing (CAD/CAM) systems were used by only a small minority, and laser dentistry, three-dimensional printing, and digital shade matching were used by almost none. Where technology was used at all, its perceived benefit skewed towards communication and workflow efficiency rather than diagnostic accuracy, suggesting that digital tools are currently valued by this workforce more as communication aids than as diagnostic or predictive instruments [6].

Age and years of practice were both significantly associated with interest in adopting new dental technologies, with more experienced practitioners and, counterintuitively, a meaningful share of very early-career respondents expressing openness to future adoption, while gender and rural-versus-urban practice location showed no significant association with this interest. This pattern points towards a workforce that is broadly receptive to technology in principle but has not yet been given the infrastructural or educational scaffolding to move from receptiveness to routine use of predictive tools specifically [6]. It also suggests that any strategy for introducing predictive modelling into general dental practice cannot rely on age or urban location as a proxy for readiness; willingness to adopt appears to be distributed unevenly across the career span in a way that a simple demographic targeting strategy would miss, and any implementation plan should instead be built around actual training background and available infrastructure rather than around assumptions tied to age or years in practice.

Discussion: Bridging algorithmic capability and chairside readiness

Read together, the technical and survey evidence point to a specific kind of mismatch. The ceiling on model performance has moved substantially, from an area under the curve in the high 0.70s for logistic regression to figures approaching 0.99 for the newest three-dimensional context-aware networks [3], but this gain has been achieved largely on curated, single-centre, retrospective datasets, while practitioners who would act on such a model's output frequently lack both the implant-specific training and the digital infrastructure, including intraoral scanning and CAD/CAM integration, that these tools presuppose [6]. The interpretability trade-off compounds this problem: the strongest discriminating models are also the least transparent, which sits awkwardly against a clinician population reporting only moderate confidence in its own implantology knowledge. This is not simply a lag that will close with time on its own; the two trajectories are driven by different incentives. Publication metrics reward incremental gains in discrimination on benchmark datasets, while clinical uptake is governed by training, equipment budgets, and medico-legal comfort, and there is no automatic mechanism aligning these two sets of incentives.

The Validation Gap Is Wider Than It Appears

Much of the deep learning implant-positioning literature reports only internal validation, meaning cross-validation folds or hold-out splits drawn from the same institution, the same acquisition protocol, and often the same CBCT unit that generated the training data, which systematically overstates the accuracy a clinician should expect elsewhere. This matters specifically for the population surveyed in the adoption literature discussed above: three-quarters were in practice under five years and the majority worked in general rather than specialist practice [6], so the case mix they see, spanning variable bone quality, medical comorbidity, and a range of implant systems, is unlikely to resemble the controlled, single-centre cohorts on which the highest performing architectures were trained and tested. A model validated only internally, then deployed into this more heterogeneous setting, is exposed to spectrum bias and data heterogeneity problems noted throughout the comparative literature [3,7]. Closing this gap requires external validation cohorts that deliberately include lower-volume, non-tertiary, and rural practice settings, drawing on a range of CBCT manufacturers and acquisition protocols, rather than treating this heterogeneity as a deployment problem to be solved only after publication.

Interpretability as a Precondition, Not a Refinement

The inverse relationship between predictive accuracy and interpretability recurs across every model family discussed in this review: logistic regression is the most transparent and the weakest discriminator, while the three-dimensional context-aware transformer networks are the strongest discriminators and the least transparent. In fields with a mature, highly specialised workforce, this trade-off is sometimes tolerated on the grounds that end-users can be trained to trust a validated black box, an argument that carries real weight in, for example, specialist radiology practice with dedicated training pathways. That assumption does not transfer cleanly to the surveyed population, where implantology-specific training was inconsistent and self-rated knowledge clustered around moderate rather than extensive [6]. For this workforce, an opaque high-accuracy recommendation is arguably less useful than a moderately accurate but explainable one, since the clinician has fewer independent grounds to sanity check an unexplained output before it reaches the patient. Explainable artificial intelligence techniques, including SHapley Additive exPlanations (SHAP)-style feature attribution for tabular predictors such as bone density and implant stability quotient, and saliency or attention map visualisation for CBCT-based positioning networks, should be treated as a reporting requirement alongside accuracy and area under the curve, not as an optional appendix included only when convenient. A secondary benefit of this approach is educational: an interpretable output doubles as a teaching aid for the early-career, variably trained clinicians the survey describes, narrowing the underlying knowledge gap while still supporting a specific treatment decision in real time.

Infrastructure Precedes Intelligence

A recurring assumption in the deep learning implant literature is that CBCT acquisition, digital impressions, and CAD/CAM-integrated planning software are already part of the clinical workflow the model will enter. The survey data complicate this assumption considerably: digital radiography was common among respondents, but intraoral scanners and CAD/CAM systems were each used by fewer than one in 10 respondents, and laser dentistry, three-dimensional printing, and digital shade matching were used by close to none [6]. A vision-transformer implant-positioning model is only actionable where a CBCT scan and a compatible planning pipeline already exist; in a practice without an intraoral scanner or a digital planning workflow, the more relevant technology gap sits several steps upstream of anything a predictive model can address on its own. This suggests a layered rather than flat view of technology readiness, in which imaging capture, digital workflow integration, and predictive analytics form a dependency chain, so that investment in predictive modelling should be paired with, rather than substituted for, investment in the imaging and digital workflow infrastructure that such models presuppose as a precondition for use.

Sequencing Adoption Around What Clinicians Already Value

Where digital tools were in use among the surveyed population, respondents attributed their benefit overwhelmingly to enhanced patient communication and, to a lesser extent, error reduction, and rarely to improved diagnostic accuracy [6]. Rather than reading this purely as a knowledge deficit, it can be read as a signal of where trust in digital tools currently sits among this workforce, and correspondingly of where the next layer of adoption is most likely to succeed. A staged introduction, beginning with tools that visualise and communicate an existing clinical judgement, for example illustrating risk to a patient during a consent discussion, before progressing to tools that materially shape the treatment plan itself, for example an artificial intelligence-recommended implant dimension or position, follows the trust gradient the survey reveals, rather than asking practitioners to leap directly to autonomous planning recommendations they have no prior experience evaluating. This staged approach also carries a training benefit: a clinician who has used a model's output as a communication aid will, by the time that model is proposed as a genuine planning input, have already built an intuitive sense of when its outputs look right and when they do not, which is precisely the kind of calibrated trust that the interpretability discussion above suggests this workforce currently lacks.

Regulatory and Medico-Legal Considerations

A dimension that the technical literature summarised in this review addresses only implicitly is the regulatory and medico-legal environment into which any clinically deployed prediction tool must fit. Software intended to inform a diagnostic or treatment planning decision increasingly falls within the scope of medical device regulation in most jurisdictions, which typically requires evidence of both analytical validity, meaning that the software reliably measures what it claims to measure, and clinical validity, meaning that its output is meaningfully associated with the clinical outcome it is meant to predict, before the software can be marketed for clinical use. Most of the architectures summarised in Table 1 have been demonstrated in the peer-reviewed technical literature but have not, on current evidence, progressed through this kind of formal regulatory clearance process for implant planning specifically, which means that a clinician who wished to use one of these tools today would in most cases be doing so outside any established regulatory pathway, with correspondingly uncertain liability if the tool's recommendation contributed to a poor outcome.

This uncertainty interacts directly with the adoption gap described elsewhere in this review. A clinician already reporting only moderate confidence in their own implantology knowledge is, if anything, less likely to adopt a tool whose liability status in the event of an adverse outcome is unclear, particularly given that current negligence standards in most jurisdictions still assess a clinician's decision against the judgement of a reasonably competent peer rather than against an algorithm's output, meaning that deferring to a model's recommendation does not straightforwardly discharge or transfer that clinician's own professional responsibility. Reimbursement is a related and equally unresolved question: even where the underlying imaging, such as a CBCT scan, is already reimbursed as part of routine implant workup, the additional planning software or the additional practitioner time required to review and adjust an artificial intelligence-generated recommendation is not, in most current fee structures, separately reimbursed, which further weakens the economic case for adoption at the level of an individual general practice, as distinct from a well-resourced academic or specialist centre that can absorb this cost as part of a broader digital workflow investment.

Implications for Research Design

For research design specifically, these threads converge most concretely in an in vivo comparative trial, contrasting conventional versus artificial intelligence-assisted implant planning, using implant stability quotient trajectory and survival as co-primary outcomes, offering a route to the prospective, externally interpretable evidence that narrative reviews like this one must otherwise substitute with retrospective and cross-sectional data. Several design features follow from the discussion above. Recruiting sites and operators across a range of implantology training backgrounds, rather than only specialist tertiary centre operators, would let such a trial estimate an effect size more representative of the surveyed workforce, at some cost to technique consistency across the trial arms. Building an explainability layer into the artificial intelligence-assisted arm, surfacing the features or regions driving each planning recommendation, would allow operator trust and override behaviour to be captured as a secondary outcome, information that would otherwise remain invisible in a trial comparing only final implant stability quotient and survival figures. Given the low baseline implant failure rate reported throughout this literature, survival should be analysed with an event-based method rather than a simple proportion, with sample size driven by the more conservative of the implant stability quotient mean comparison estimate and the event-based survival estimate rather than by the implant stability quotient comparison alone [5]. Finally, given the regulatory and reimbursement uncertainty discussed above, such a trial would ideally capture practitioner-reported measures of trust, perceived liability, and time cost alongside its clinical outcomes, since these factors are likely to determine real-world uptake at least as strongly as the trial's primary clinical endpoints.

Limitations of This Synthesis

This discussion draws technical performance figures and survey findings from a limited number of sources rather than from a systematic, exhaustive search of the literature, and the survey evidence comes from a single institution and region, which limits how far its training and infrastructure findings can be generalised to other health systems or practice settings. The deep learning performance figures are similarly drawn from originating methodological descriptions rather than from a pooled, independently conducted meta-analysis, and, as noted in the comparative performance section above, they describe two only partly comparable tasks, tabular outcome classification on the one hand and volumetric position regression on the other, so the figures should be read as illustrative of the field's overall trajectory rather than as a definitive head-to-head ranking of model families on a single, shared task. These limitations do not undermine the central argument of this review, namely, that model capability and clinical readiness are currently advancing along different trajectories driven by different incentives, but the specific numeric gaps reported here should be treated as indicative of a broad pattern rather than as precise, directly comparable estimates [12].

## Conclusions

Predictive modelling in dental implantology has progressed from interpretable but limited regression-based tools to deep, image native architectures with markedly higher discrimination on benchmark tasks. That progress, however, has outpaced the clinical and infrastructural readiness of much of the workforce that would apply it: survey evidence indicates that implant-specific training remains inconsistent among general practitioners, digital infrastructure beyond basic radiography is limited outside specialist centres, the perceived value of existing technology centres on communication and efficiency rather than on diagnostic or predictive support, and the regulatory and liability framework surrounding clinical use of these tools remains largely undefined. Closing this gap will depend less on further increments in model accuracy and more on rigorous external validation across heterogeneous practice settings, explainable outputs treated as a reporting requirement rather than an optional extra, standardised reporting through frameworks such as TRIPOD-AI, clearer regulatory and liability guidance, and a realistic, staged approach to technology adoption that meets clinicians where their current practice, training, and infrastructure already stand rather than where the technical literature assumes them to be.

## References

[1] Mahendra J, Subbiah U, GopalaKrishnan P, Karunanidhi K, Rajendran S, Mohan S (2025). Decoding success: a five-year retrospective study of dental implant survival. J Oral Biol Craniofac Res.

[2] Joshi M, Kaushik M, Rana M, Kaushik S (2026). Conventional and basal implantology: a narrative review of current evidence, clinical decision-making, and future perspectives. Cureus.

[3] Rademakers FE, Biasin E, Bruining N (2025). CORE-MD clinical risk score for regulatory evaluation of artificial intelligence-based medical device software. NPJ Digit Med.

[4] Gao S, Wang X, Xia Z, Zhang H, Yu J, Yang F (2025). Artificial intelligence in dentistry: a narrative review of diagnostic and therapeutic applications. Med Sci Monit.

[5] Tonin BS, Fu J, Souza LF, Özcan M (2026). A digital translational framework for biomaterials validation: from evidence- to model-based dentistry. Trans Dent Res.

[6] Neji G, Gasparro R, Tlili M (2025). AI-powered predictive models in implant dentistry: planning, risk assessment, and outcomes. J Clin Med.

[7] Brizuela-Velasco A, Álvarez-Arenal Á, Pérez-Pevida E, Bellanco-De La Pinta I, De Llanos-Lanchares H, González-González I, Larrazábal-Morón C (2021). Logistic regression analysis of the factors involved in the failure of osseointegration and survival of dental implants with an internal connection and machined collar: a 6-year retrospective cohort study. Biomed Res Int.

[8] Burlea ȘL, Buzea CG, Nedeff F (2025). Deep learning analysis of CBCT images for periodontal disease: phenotype-level concordance with independent transcriptomic and microbiome datasets. Dent J (Basel).

[9] Daldal M, Baybars SC, Baydoğan MP, Tuncer SA (2025). Classification of apical openness using vision transformer: a comparative approach with expert decisions. J Imaging Inform Med.

[10] Yang X, Li X, Li X, Wu P, Shen L, Deng Y (2024). ImplantFormer: vision transformer-based implant position regression using dental CBCT data. Neural Comput Applic.

[11] Yang X, Xie J, Li X, Li X, Li X, Shen L, Deng Y (2023). TCEIP: text condition embedded regression network for dental implant position prediction. Medical Image Computing and Computer Assisted Intervention - MICCAI 2023.

[12] Khan R, Khan S, Almohaimeed HM, Almars AI, Pari B (2025). Utilization, challenges, and training needs of digital health technologies: perspectives from healthcare professionals. Int J Med Inform.

